# Retraction Note: Nutrient strengthening and lead alleviation in *Brassica Napus* L. by foliar ZnO and TiO_2_-NPs modulating antioxidant system, improving photosynthetic efficiency and reducing lead uptake

**DOI:** 10.1038/s41598-026-46179-5

**Published:** 2026-03-30

**Authors:** Adiba Khan Sehrish, Shoaib Ahmad, Sarah Owdah Alomrani, Azeem Ahmad, Khalid A. Al-Ghanim, Muhammad Ali Alshehri, Arslan Tauqeer, Shafaqat Ali, Pallab K. Sarke

**Affiliations:** 1 State Key Laboratory of Pollution Control and Resource Reuse, School of the Environment, Nanjing University, Nanjing, 210023 Jiangsu China; 2 Department of Biology, College of Science and Arts, Najran University, 66252 Najran, Saudi Arabia; 3 Soil and Water Chemistry Laboratory, Institute of Soil and Environment Sciences, University of Agriculture, Faisalabad, Pakistan; 4 Department of Zoology, College of Science, King Saud University, 11451 Riyadh, Saudi Arabia; 5 Department of Biology, Faculty of Science, University of Tabuk, 71491 Tabuk, Saudi Arabia; 6 School of Modern Engineering and Applied Sciences, Nanjing University, Nanjing Jiangsu, 210023 China; 7 Department of Environmental Science, Government College University, Faisalabad, Faisalabad, 38000 Pakistan; 8 Department of Biological Sciences and Technology, China Medical University, Taichung, 40402 Taiwan; 9 Environmental Studies Department, University of California Santa Cruz, Santa Cruz, CA 95060 USA

Retraction of: *Scientific Reports* 10.1038/s41598-024-70204-0, published online 21 August 2024

The Editors have retracted this Article.

After publication, the Editors were contacted by Pallab K. Sarke who stated that he had played no role in the creation of this article. The Editors requested the underlying raw data. Some of the key data provided did not contain metadata that would allow for its verification. Additionally, discrepancies were noted between the description of experimental methods and the reported results. Finally, the authorship of this paper could not be verified. The Editors have therefore lost confidence in the integrity of the Article.

None of the Authors responded to the Editor’s correspondence about this retraction.

